# Transfer Learning for Heterocycle Retrosynthesis

**DOI:** 10.1021/acs.jcim.4c02041

**Published:** 2025-07-29

**Authors:** Ewa Wieczorek, Joshua W. Sin, Sara Tanovic, Matthew T. O. Holland, Liam Wilbraham, Victor Sebastián-Pérez, Anthony Bradley, Dominik Miketa, Paul E. Brennan, Fernanda Duarte

**Affiliations:** † Chemistry Research Laboratory, 12 Mansfield Road, Oxford OX1 3TA, U.K.; ‡ Alzheimer’s Research UK Oxford Drug Discovery Institute, Centre for Artificial Intelligence in Precision Medicine, Centre for Medicines Discovery, Nuffield Department of Medicine, 6396 University of Oxford, Oxford OX3 7FZ, U.K.; § 422230 Exscientia plc, The Schrödinger Building Oxford Science Park, Oxford OX4 4GE, U.K.

## Abstract

Heterocycles are important scaffolds in medicinal chemistry that can be used to modulate the binding mode as well as the pharmacokinetic properties of drugs. The importance of heterocycles has been exemplified by the publication of numerous data sets containing heterocyclic rings and their properties. However, those data sets lack synthetic routes toward the published heterocycles. Consequently, novel and uncommon heterocycles are not easily synthetically accessible. While retrosynthetic prediction models could usually be used to assist synthetic chemists, their performance is poor for heterocycle formation reactions due to low data availability. In this work, we compare the use of four different transfer learning methods to overcome the low data availability problem and improve the performance of retrosynthesis prediction models for ring-breaking disconnections. The mixed fine-tuned model achieves top-1 accuracy of 36.5%, and, moreover, 62.1% of its predictions are chemically valid and ring-breaking. Furthermore, we demonstrate the applicability of the mixed fine-tuned model in drug discovery by recreating synthetic routes toward two drug-like targets published in 2023. Finally, we introduce a method for further fine-tuning the model as new reaction data becomes available.

## Graphical abstract

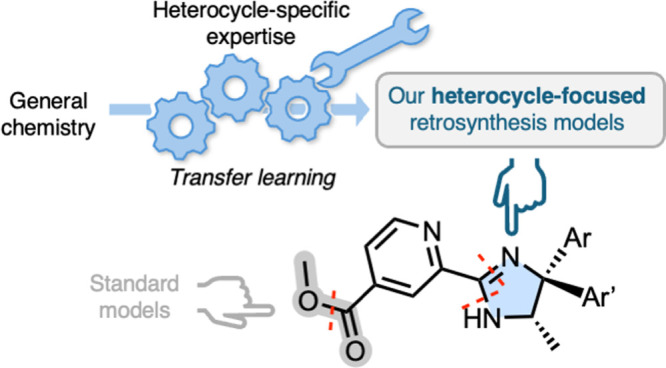

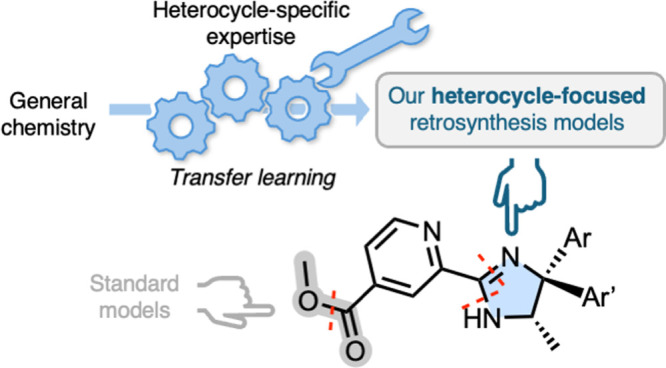

## Introduction

Retrosynthesis, the iterative process of breaking down a molecule into simpler precursors, has traditionally been the domain of expert organic chemists.^1^ However, even for experienced chemists, this approach presents challenges due to the vast chemical space of potential transformations and the incomplete understanding of reaction mechanisms and their dependence on reaction conditions. To overcome these challenges, efforts have persisted since the 1970s to integrate computation into synthetic planning by developing Computer-Aided Synthesis Planning (CASP) tools, with one of the earliest examples being the Logic and Heuristics Applied to Synthetic Analysis (LHASA) by Pensak and Corey.^2^ Despite numerous attempts, CASP tools had limited success until recently.^3^

Significant progress in CASP tools has occurred in the past decade,^4^ driven by advances in machine learning (ML) methodologies and the availability of chemical data sets, such as Lowe’s US Patents Office (USPTO) reaction extracts.^5^ Following the seminal work by Segler et al.^6^ on the use of neural networks and search algorithms in the 3N-MCTS CASP tool, there has been a proliferation of new ML models for retrosynthesis prediction. These models can be broadly classified into two categories: template-based^6–9^ and template-free methods.^10–14^ Template-based methods rely on predefined reaction rules extracted from data sets, where algorithms match a target molecule with predefined templates. CASP tools utilizing such models include ASKCOS,^7^ AiZynthFinder,^8^ and Retro*.^9^ In contrast, template-free methods, such as graph-based^13,14^ or sequence-to-sequence^10–12^ (seq2seq) approaches, bypass the use of an external template database by directly training on raw reaction data. While early seq2seq models were based on long–short-term memory networks (LSTMs),^12^ the breakthrough in seq2seq reaction prediction came when Schwaller et al. applied the transformer model^15^ commonly used in natural language processing (NLP) for forward reaction prediction, creating the Molecular Transformer.^16^ In this case, reaction prediction is treated as a translation problem using Simplified Molecular Input Line Entry System (SMILES)^17^ strings to represent the chemical transformation. Since then, seq2seq retrosynthesis prediction models have shown high accuracies on public benchmarking test sets, with the Augmented Transformer^11^ achieving 46.2% top-1 reactant accuracy on the USPTO-full data set.^18^ The recent developments have led to transformers emerging as a premier architecture for retrosynthesis planning utilized in platforms such as IBM RXN.^10^

Despite the high efficacy of CASP tools on general reaction data sets, predicting retrosynthetic disconnections for specific, less prevalent areas of chemistry remains a significant challenge due to data set bias.^19,20^ Heterocycle formation reactions are an example of underrepresented reaction classes, accounting for only 5% of reported chemical reactions in the USPTO data set.^19^ However, heterocycles are key motifs in drug design, with 85% of the top 200 best-selling small-molecule drugs of 2022 featuring heterocyclic rings.^21^ Through bioisosteric replacement of the rings with other heterocycles, pharmacokinetic and toxicological properties of lead compounds can often be improved.^22–24^ Although numerous virtual libraries document theoretically synthesizable heterocyclic scaffolds,^25^ synthetic pathways toward novel heterocycles remain underexplored, with the focus in medicinal chemistry being on ring derivatization rather than ring formation.^26,27^ Enhancing the prediction capacity of CASP tools for reactions forming these crucial chemical motifs could stimulate the exploration of novel heterocyclic molecules, potentially fueling new therapeutic breakthroughs.

This work aims to enhance the performance of CASP tools for heterocycle retrosynthesis by combining seq2seq models and transfer learning, where knowledge learned from one task is used to boost the performance on a related task (Figure 1). Two transfer learning approaches, fine-tuning and multitask learning, have been previously successfully applied for the forward reaction prediction of carbohydrate reactions^20^ and Heck reactions,^28^ as well as forward and retrosynthesis prediction of enzymatic reactions^29,30^ (Figure 2a). However, both come with limitations. For example, in the reported examples, fine-tuning showed a quick training time and increased accuracy for reactions of interest but showed low performance for common reactions. Conversely, multitask learning maintained good performance across reaction types but required longer training time, making it less suitable for frequent retraining as new data emerges. To address these limitations, we evaluate mixed fine-tuning^31^ and ensemble decoding,^32^ previously proven effective in language translation but not yet used in retrosynthesis prediction (Figure 2b). We compare those methods to the template-based approach reported by Thakkar et al., “Ring Breaker”, specifically for ring-forming reaction prediction.^19^ To train these models, we use a large data set of all reaction types based on USPTO (“*General*”) and a smaller data set of just heterocycle formations (“*Ring*”). Our results show that the *mixed fine-tuned* model is the best for multistep retrosynthesis, with a 10% increase in accuracy over the baseline for heterocycle formations and similar performance for other reactions. We demonstrate the applicability of the *mixed-fine-tuned* model by predicting retrosynthetic routes for two recently published heterocycle-containing drug-like targets. Finally, we test the model on recently developed heterocycle formations and demonstrate how it can be further fine-tuned to improve its accuracy with these new datapoints.

**Figure 1:**
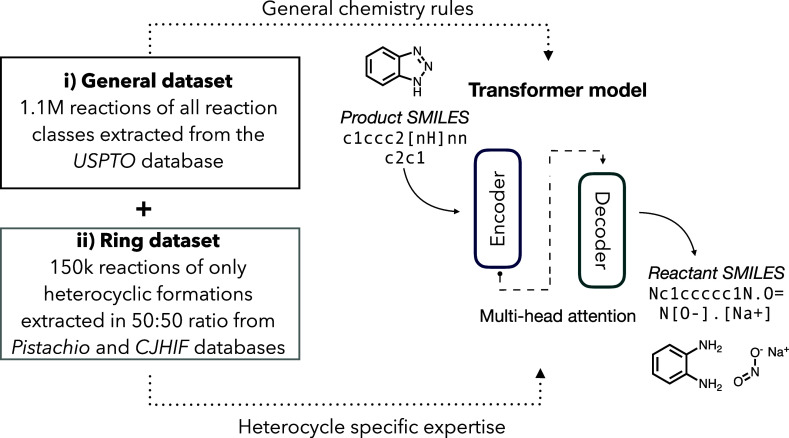
Utilization of general (i) and domain-specific (ii) data in transfer learning approaches for sequence-to-sequence retrosynthesis prediction.

**Figure 2:**
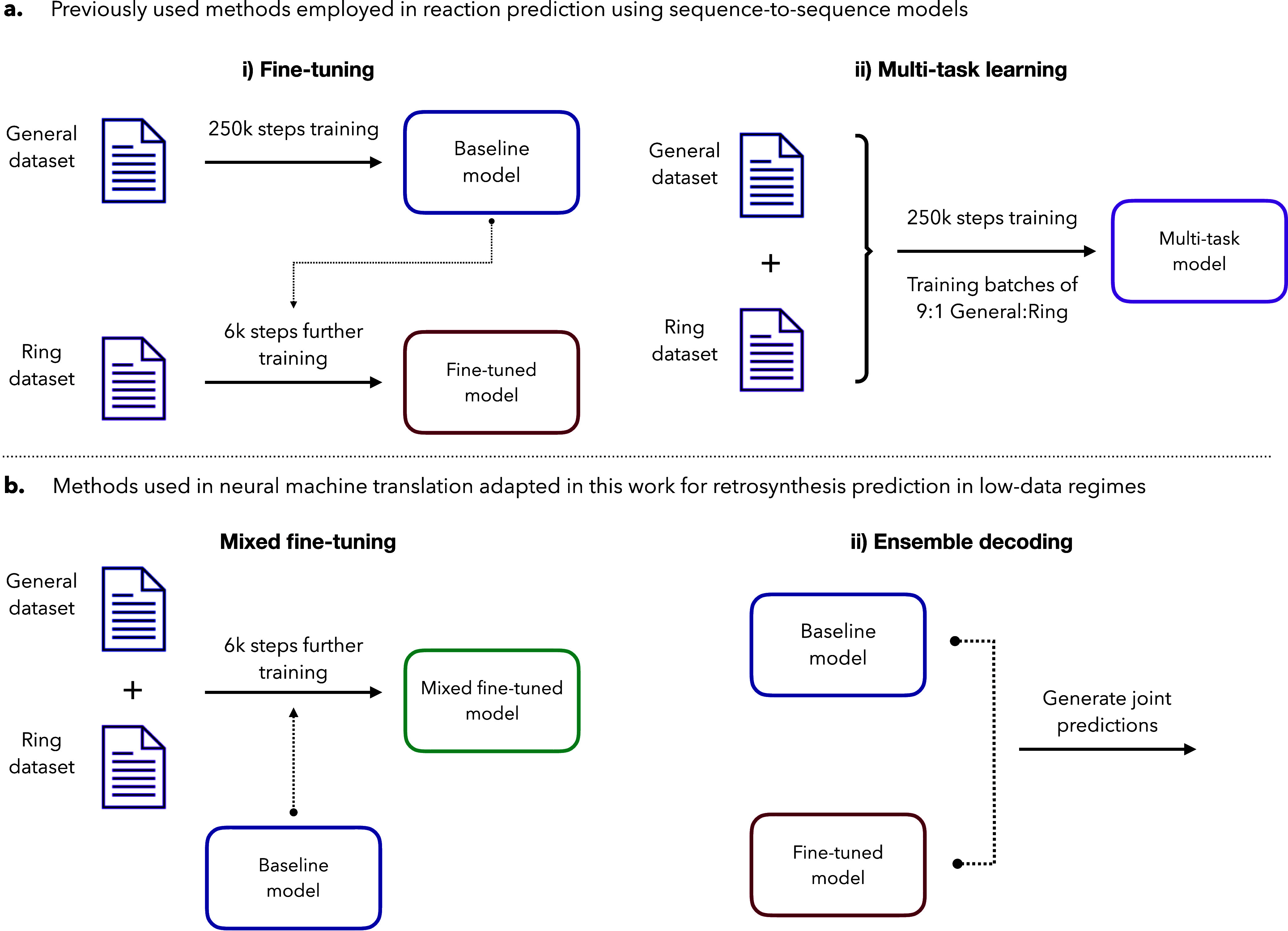
Overview of transfer learning methods used in this work for heterocycle retrosynthesis prediction. (a) Methods previously used for forward reaction prediction and retrosynthesis. Fine-tuning consists of training a baseline model on a large data set of all reaction classes, which is then fine-tuned on a smaller data set of only reactions of interest. In multitask learning, the model is trained on both data sets at the same time. (b) Methods only used in NLP tasks. In mixed fine-tuning, the *baseline* model is fine-tuned on both data sets. In ensemble decoding, the prediction is made jointly with the *baseline* and *fine-tuned* models. For all relevant methods, the baseline model is shown in blue, the *fine-tuned* model in red, the *mixed fine-tuned* model in green, and the *multitask* model in purple.

## Methods

### Data Sets

In this study, we utilized the USPTO data set preprocessed by Pesciullesi et al.,^20^ which is henceforth referred to as the *General* data set. Additionally, we curated a data set of 165,216 ring formation reactions, referred to here as the *Ring* data set, comprising about 80k reactions extracted from academic journals (CJHIF data set^33^) and 80k reactions from additional patent data (Pistachio data set, accessed 28th June 2022, version 2022Q1).^34^ The creation of the *Ring* data set is described in more detail in Supporting Information S3. Visualizations of the chemical space of the data sets are included in Supporting Information S4, showing that ring-breaking reactions occupy distinct areas of the chemical space.

The *Ring* data set was split into train, validation, and test sets with a 90:5:5 ratio based on the Tanimoto similarity of reaction products^35^ using DeepChem.^36^ The *General* data set splitting was retained from the work of Pesciullesi et al.^20^ Additionally, we performed a random split of the *Ring* data set and trained the mixed fine-tuned model on the randomly split data set to assess the effect of data set splitting (Supporting Information S7).

### Retrosynthesis Prediction Models

We trained the single-step retrosynthesis prediction models based on the seq2seq Transformer architecture using the OpenNMT-py package.^37^ All hyperparameters used here are provided in the Supporting Information S1 and are based on the work of Pesciullesi et al.^20^ The *baseline* model was trained on the *General* data set, while the *ring-only* model was trained on the *Ring* data set. As fine-tuning and multitask learning have been previously used for reaction prediction, we adopted the parameters previously reported for these models. For the *multitask* model, we used a data set weight ratio of 9 (*General*):1 (*Ring*) (Figure 2a­(ii)). For the *fine-tuned* model, the number of fine-tuning steps was set to 6000 (Figure 2a­(i)). For mixed fine-tuning (Figure 2b­(i)), a 1:1 data set weight ratio and 6000 fine-tuning steps were chosen after a benchmark (Supporting Information S6). Ensemble decoding was performed with built-in OpenNMT-py functionality using the *fine-tuned* model and the *baseline* model (Figure 2b­(ii)).

Furthermore, we trained a single-step template-based retrosynthesis prediction model on only ring-forming reactions based on the approach introduced by Thakkar et al. in “Ring Breaker”,^19^ which used the improved ring-breaking template extraction approach from AiZynthTrain.^38^ This template-based model is trained exclusively on ring-formation reactions and, in practice, is used in conjunction with another model of the same architecture trained on a broader set of reaction types.

Our data set comprised reactions from the *Ring* data set and ring formations extracted from the *General* (USPTO^5^) data set. Atom mapping of reaction data was conducted using RXNMapper,^39^ and reaction templates were subsequently extracted using AiZynthTrain. The default settings of AiZynthTrain were used to train the models, and a custom split was enforced to maintain consistent data splits across the experiments.

To adapt the trained single-step retrosynthesis prediction models to multistep route planning tools, we used a neural-based A* search algorithm based on Retro*.^9^ Multistep route planning tools were constructed for both the baseline and mixed fine-tuned single-step models. The stock molecule database chosen was eMolecules (version accessed with Retro* code implementation from Chen et al.,^9^ 11th January, 2019).

### Model Evaluation Metrics

The single-step retrosynthesis prediction models were evaluated on both the *General* and *Ring* test sets using metrics based on top-*N* accuracy and round-trip accuracy.^10^ For both the *Ring* and the *General* test sets, we calculate reactant-only accuracy, where the prediction is considered accurate if all of the ground truth reactants are present. While the reactants and reagents in the *Ring* data set were separate, for the *General* test set, the precursors were assigned as either reactants or reagents after atom mapping with rxnmapper.^39^ We also consider the round-trip accuracy^10^ of the suggested disconnections, which represent the “chemical validity” of predictions, i.e., what proportion of predicted reactant sets are expected to produce the desired product. Additionally, we introduce a new metric: the ring-breaking round-trip accuracy, calculated only for the “Ring” data set. A disconnection is considered to be ring-breaking round-trip-accurate when it is round-trip-accurate, and the number of rings in the product is higher than in predicted reactants. In this way, we consider not only whether the prediction is chemically valid but also whether it involves a ring disconnection, i.e., the reaction type we’re aiming to improve the model’s performance for.

All metrics reported in the main text are for top-1 predictions. However, metrics for the top-3 and top-5 predictions are available in the Supporting Information S8. A more detailed explanation of the metrics can be found in Supporting Information S5.

### Further Fine-Tuning

We extracted a set of 1475 heterocycle formations from 47 scientific publications from 2022 reporting new methodologies for heterocycle synthesis (Supporting Information S10). This data set (referred to as the *Recent* data set) was split randomly into train, validation, and test sets with a ratio of 80:10:10. Further fine-tuning was carried out using the mixed fine-tuning approach, starting from the *mixed fine-tuned* model and training it for 6000 steps on the *General*, *Ring*, and *Recent* data sets with a 4:4:1 data set weight ratio.

## Results

### Optimization of the Single-Step Retrosynthesis Model

#### Comparative Analysis of Transfer Learning Approaches

We commenced our study by comparing the performance of different transfer learning approaches, focusing on methods previously used for chemical reaction prediction (i.e., multitask learning and fine-tuning) and methods employed in the NLP domain (mixed fine-tuning and ensemble decoding) (Figure 2). This comparison was conducted on the *Ring* test set to assess their performance in predicting ring-breaking reactions against the *baseline* model trained on the *General* data set and the *ring-only* model trained on the *Ring* data set (Figure 3A). In addition to reactant accuracy, we also evaluated whether the prediction was chemically valid and corresponded to a ring-breaking reaction. This identifies predictions that differ from the ground truth disconnection present in the test set but still disconnect the ring.

**Figure 3:**
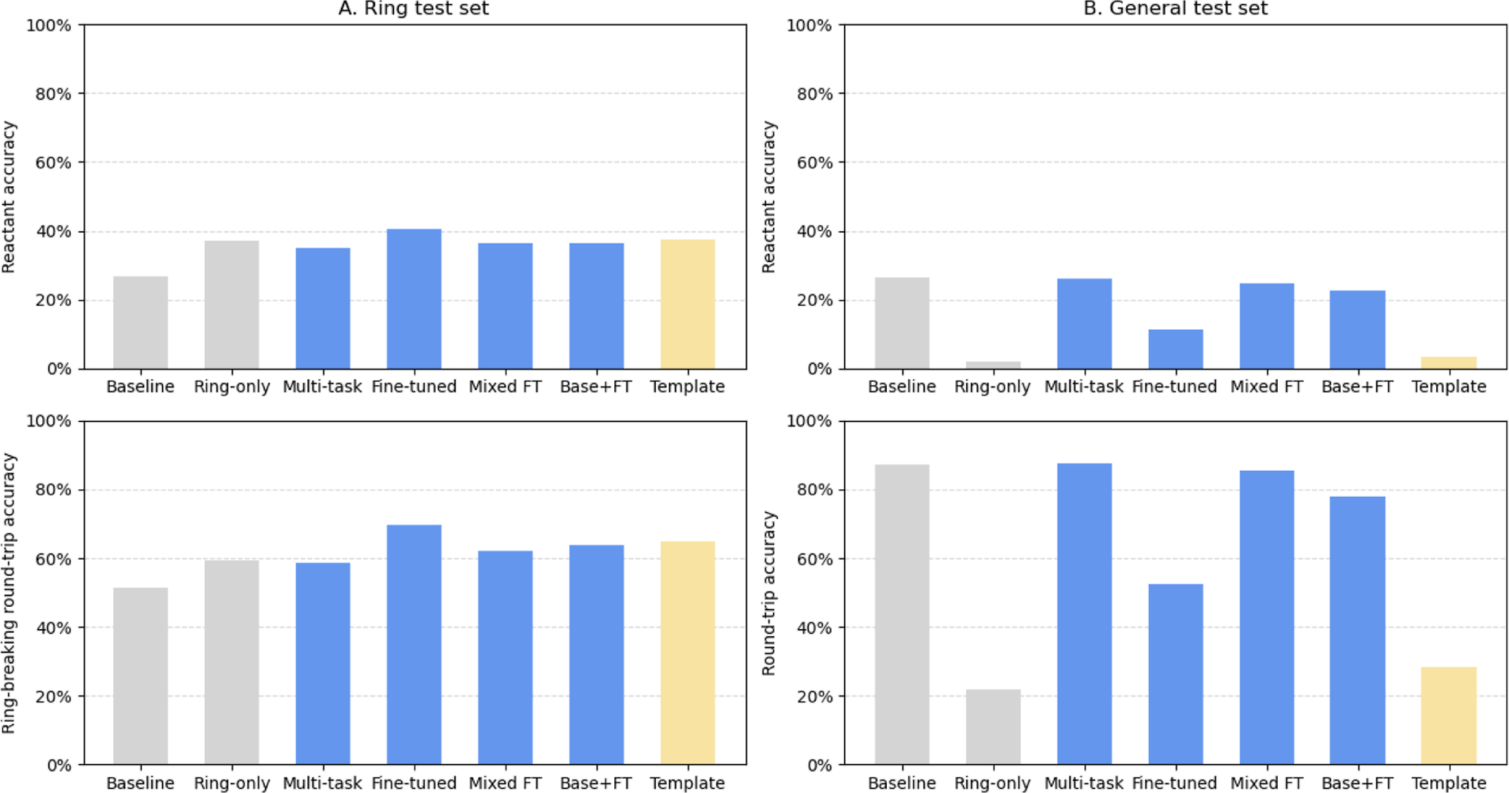
Comparison of the model performance on the (A) *Ring* and (B) *General* test sets. For the *Ring* test set, top-1 reactant accuracy and proportion of valid ring-breaking top-1 predictions are shown. For the *General* test set, top-1 reactant accuracy and round-trip accuracy are shown. Different architectures are distinguished by color: the models trained on a single data set (*baseline* and *ring-only*) are in gray; *multitask, fine-tuned, mixed fine-tuned* (Mixed FT) models and ensemble decoding (Base+FT) are in blue; and the *template-based* (Template) model is shown in yellow.

Our results show that on the *Ring* test set, the *fine-tuned* model outperforms the other approaches, achieving a top-1 reactant accuracy of 40.5% (Figure 3A). Moreover, 69.5% of all its top-1 predictions are chemically valid and correspond to ring-breaking reactions. The three other approaches also show improvement over the *baseline* model with top-1 reactant accuracies of around 36% and 62% valid ring-breaking top-1 predictions. However, they perform similarly to the *ring-only* model, which achieves reasonable accuracy at 37.2% top-1 reactant accuracy. This comparatively high accuracy can most likely be achieved due to the larger transfer data set size than that used in previous studies (160k reactions here vs 20k reactions used previously).^20^ Although the improvement over the *baseline* is not as high (13.6% increase in accuracy for the *fine-tuned* model) compared to previous studies for carbohydrate reactions (27.0%)^20^ and Heck reactions (28.6%),^28^ two key aspects should be noted. First, these studies used transfer learning for forward reaction prediction, an easier task than retrosynthesis, only having one “correct” answer. Second, heterocycle formations are a much larger and more diverse class of reactions than Heck or carbohydrate reactions, making it more difficult for the model to learn the different reactivities.

Interestingly, even though each of our approaches increases the proportion of top-1 valid ring-breaking predictions by at least 7% when compared to the *baseline* model, the same trend is not observed when considering just the top-1 round-trip accuracy of the predictions (Supporting Information S8). For example, for the *mixed fine-tuned* model, the ring-breaking round-trip accuracy increases by over 10%, while the round-trip accuracy decreases by 1%. The same trend can be observed for all other approaches apart from the *multitask* model, where the round-trip accuracy increases but not as much as the ring-breaking round-trip accuracy (Supporting Information S8). This indicates that the main improvement between the various models trained using transfer learning and the *baseline* model is in the type of disconnection suggested, i.e., ring-breaking versus more common reaction types, and not in turning chemically invalid disconnections into valid ones. It also suggests that while the molecules in the *Ring* test set were synthesized using ring formation reactions, there are other chemically viable disconnections available.

Indeed, comparing the predictions of the *baseline* and *mixed fine-tuned* models revealed that the former often suggested more common reaction types, such as functional group interconversions (FGIs) or protection/deprotections, instead of the ground-truth heterocycle formation predicted by the *mixed fine-tuned* model (Figure 4). For instance, in example Figure 4A, the *mixed fine-tuned* model correctly identifies a click reaction to generate the triazole from two fragments of similar complexity. In contrast, the *baseline* model suggests only a more trivial N-alkylation reaction. Similarly, for Figure 4B, the *mixed fine-tuned* model suggests a condensation reaction to form the central benzimidazole ring, while the *baseline* model suggests a functional group interconversion, which would be more suitable earlier in the synthetic route. In Figures 4C and 4D, the *baseline* model predicts simple halogenation reactions rather than ring disconnections. Interestingly, although the *mixed fine-tuned* model’s prediction is accurate for Figure 4D, it was not counted as round-trip accurate due to the forward model predicting a condensation reaction with both the carboxylic acid and the nitro group instead of just a single condensation with the former. This highlights a limitation of metrics based on round-trip accuracy, where the model’s prediction is only assessed by another model that is not 100% accurate instead of comparing the prediction to those reported in the literature or assessed by skilled organic chemists. Finally, in Figure 4E, the *mixed fine-tuned* model correctly predicts the disconnection of indazole, while the *baseline* model suggests a Boc protection of the nitrogen without simplifying the molecule. While the ability of the model to suggest protection reactions is notable, as they are crucial parts of synthetic routes, this specific protection is unnecessary and might lead the model to predict a cycle of protection/deprotection reactions, preventing further disconnections of the molecule.

**Figure 4:**
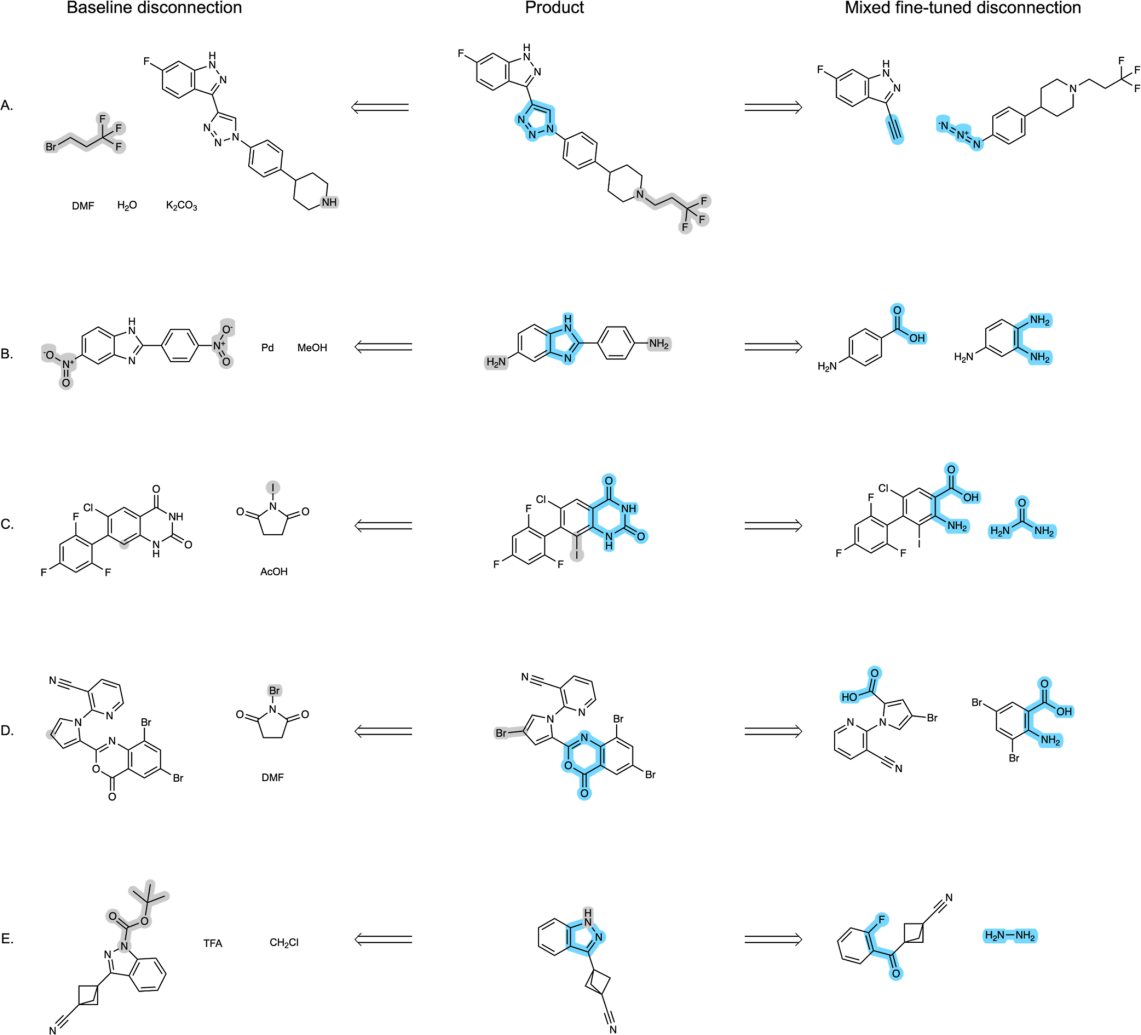
Example top-1 predictions of the *mixed fine-tuned* and *baseline* models for *Ring* test set molecules. For all the examples shown, the *mixed fine-tuned* prediction was accurate, while the *baseline* prediction was valid but not ring-breaking. The disconnections suggested by the *mixed fine-tuned* model are highlighted in blue, while the disconnections suggested by the *baseline* model are highlighted in gray.

When tested on the *General* test set, the models exhibit almost the opposite trend (Figure 3B). Performance of the *fine-tuned* model drastically decreases compared to the *baseline* model, with the top-1 reactant accuracy dropping from 26.4% to 11.4% and top-1 round-trip accuracy from 87.4% to 52.6%. The performance of the *ring-only* baseline is even poorer, with only 2.0% top-1 reactant accuracy and 21.8% round-trip accuracy. Meanwhile, the metrics for the *mixed fine-tuned* and *multitask* models only change marginally, dropping by at most 2%. Ensemble decoding falls in between, with a top-1 reactant accuracy of 22.7% and round-trip accuracy of 77.9%. The drop in performance observed with the *fine-tuned* model can most likely be attributed to catastrophic forgetting,^40^ the tendency of NNs to forget previously learned information when trained on new data. This drop can be disregarded if the model is intended for only one-step ring disconnection. However, it becomes problematic for multistep retrosynthesis as the fine-tuned model will not be able to disconnect the linear intermediates obtained after disconnecting the ring. In that case, either the *mixed fine-tuned* or *multitask* models would be more suitable.

Considering time and resources, mixed fine-tuning appears preferable due to its 40 times shorter training time and comparable performance to multitask learning, especially if planning to frequently retrain the model as new data becomes available. Ensemble decoding employs two models to make the prediction, and therefore, it takes longer to calculate than the other three methods.

Overall, both multitask learning and mixed fine-tuning show improved performance for ring-breaking disconnections while retaining the ability to predict other reaction classes, with mixed fine-tuning being preferable due to shorter training time. While the *fine-tuned* model performs best for heterocycle disconnections, it is not suitable for multistep retrosynthesis due to catastrophic forgetting. Ensemble decoding ranks in the middle, not being as good at ring disconnections as the *fine-tuned* model, but also performing worse for other reaction classes than the *mixed fine-tuned* model. Due to this, we perform all further experiments and comparisons with the *mixed fine-tuned* model, as the most versatile and best-performing one.

#### Comparison to the Template-Based Model

The *mixed fine-tuned* model was further benchmarked against “Ring Breaker”,^19,38^ the template-based model trained specifically for heterocycle retrosynthesis. To allow for a fair comparison, we retrained “Ring Breaker” with our additionally extracted ring formation data, using the whole *Ring* data set and ring formation reactions from the *General* data set. We compared the performance of the *mixed fine-tuned* model to this ring-breaking specific template-based model.

In terms of reactant accuracy, both the *mixed fine-tuned* and the template-based models have similar top-1 reactant accuracies (Figure 3A), with the template-based model’s reactant accuracy being slightly higher. However, the *mixed fine-tuned* model has significantly higher top-1 round-trip accuracy. These trends remain consistent across top-3 and top-5 predictions (Supporting Information S8). Moreover, the round-trip accuracies for the template-based model decrease rapidly from top-1 to top-5, from 64.8% to 52.4%, while the *mixed fine-tuned* model maintains high round-trip accuracy from top-1 (74.6%) to top-5 (71.8%) (Table 1). The *mixed fine-tuned* model also suggests a comparable (for top-1 predictions) or higher (for top-3 and top-5) overall proportion of chemically valid ring-breaking disconnections (defined in Methods), with 52.4% for the *mixed fine-tuned* model compared to 30.8% for the template-based model in the first 5 predictions (Supporting Information S8). Additionally, the *mixed fine-tuned* model maintains considerable accuracy on the *General* test set, while the template-based model achieves a low top-1 reactant accuracy of 3.2%.

**Table 1:** Comparison of the Template-Based Model to the Mixed Fine-Tuned Model^a^

	mixed fine-tuned model			template-based model		
metric	top-1 (%)	top-3 (%)	top-5 (%)	top-1 (%)	top-3 (%)	top-5 (%)
round-trip accuracy	74.6	73.3	71.8	64.8	58.1	52.4
inadmissible predictions	0.5	0.7	0.8	10.2	23.6	35.4

a Top-*N* round-trip accuracy refers to the proportion of predictions within the first *N* predictions for the test set considered chemically valid. The proportion of inadmissible predictions refers to the percentage of predictions in the first *N* predictions for the test set that did not output a viable SMILES string.

Furthermore, we observe that the template-based model generates a larger proportion of nonadmissible predictions of “None”, with 35.4% of top-5 predictions being inadmissible, compared to only 0.8% of the *mixed fine-tuned* model’s predictions corresponding to invalid SMILES strings (Table 1). For the template-based model, the increase in the proportion of inadmissible predictions between top-1 and top-5 correlates with the decrease in round-trip accuracy, indicating that the low round-trip accuracy is partially due to the model’s inability to apply multiple templates to one molecule. Hence, it is likely that the *mixed fine-tuned* model learns a wider range of chemistry than the template-based model, which is limited in diversity when it comes to disconnection strategies.

Overall, our results demonstrate that the *mixed fine-tuned* model significantly outperforms the template-based model in round-trip accuracy, suggesting more diverse disconnections for both general and ring-breaking disconnections, making it the preferred choice for multistep retrosynthesis, as discussed in the following section. However, it is important to note that the forward reaction prediction model used for calculating round-trip accuracy has the same architecture as the *mixed fine-tuned* model and is trained on the same reaction data (but with reversed labels). This could be biasing the metric toward the *mixed fine-tuned* model and mean that the difference in round-trip accuracy between the *mixed fine-tuned* model and the template-based model is not as significant as it seems. A more objective way of calculating metrics such as round-trip accuracy could be to use a different model to predict reaction viability instead of the forward reaction prediction model; however, we were not able to train such a model for this work due to a lack of negative reaction data.

### Mixed Fine-Tuned Multistep Model

To assess the practical use of the *mixed fine-tuned* model in synthesis planning for drug-like targets, we constructed a multistep retrosynthesis prediction tool using neural-guided A* Search, based on the algorithm used in Retro*. The two drug-like targets included **CZS-035** and **ADD** (Figure 5), for which syntheses were reported in 2023. The exact reactions employed in these syntheses are therefore absent in our training set, which contains reactions from patents and the literature up to 2022. For comparison, we also built an analogous multistep retrosynthesis tool employing the baseline single-step model, maintaining identical search settings.

**Figure 5:**
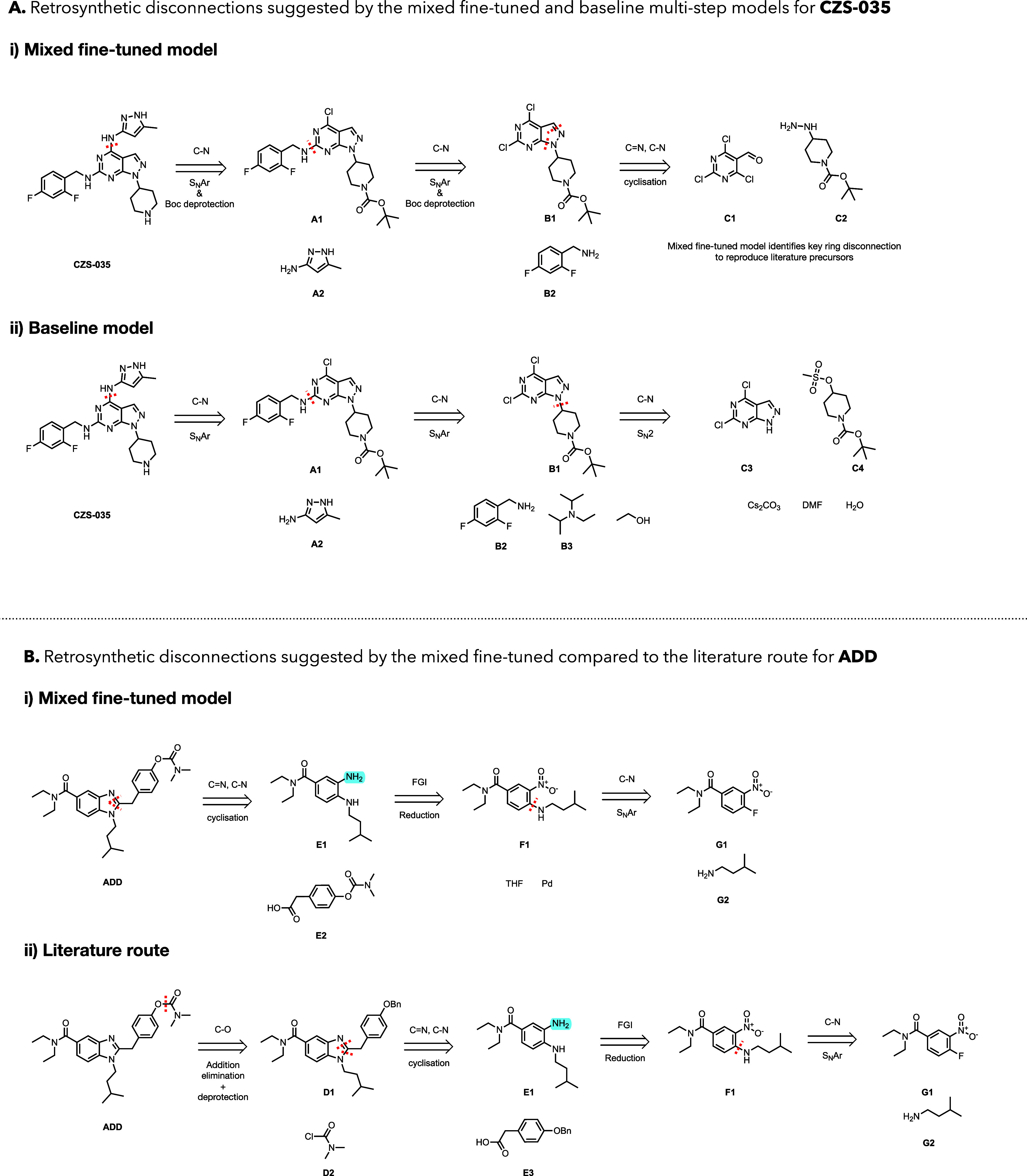
Example synthetic routes found by the *mixed fine-tuned* model for molecules of clinical interest. (A) Comparison of the retrosynthetic routes for **CZS-035** predicted by (i) *mixed fine-tuned* and (ii) *baseline* models. (B) The retrosynthetic route for **ADD** (i) predicted by the *mixed fine-tuned* model compared to (ii) the literature route. The *baseline* model failed to predict a complete route for this compound.

The first case study, **CZS-035**, is a ligand for polo-like kinase 4 (PLK4) and a warhead component used to synthesize a therapeutic PROTAC for breast cancer treatment, discovered by Sun et al.^41^ (Figure 5A). Both the *baseline* and *mixed fine-tuned* multistep models successfully identify retrosynthetic routes for **CZS-035** from purchasable precursors in our stock molecule database. Both models accurately reproduce the protection of nitrogen with Boc (**A1)** as seen in the literature synthesis.^41^ Both models also correctly identify the two S_N_Ar disconnections used in the literature to reproduce **B1** and **B2**. However, the *mixed fine-tuned* model uniquely identifies the final ring disconnection of pyrazole in **B1** to **C1** and **C2**, which aligns with the literature approach. In contrast, the *baseline* model suggests the more complex and more expensive pyrazolopyrimidine **C3** as the final purchasable precursor. This result showcases the enhanced performance of the *mixed fine-tuned* model for predicting key ring disconnections for multistep routes, overcoming catastrophic forgetting, and correctly identifying all non-ring-breaking disconnections of **CZS-035**. We note that the ability of seq2seq models over template-based models to simultaneously suggest protections and S_N_Ar disconnections in different sites, as in **A1**, is a unique advantage.

The second case study was **ADD** (compound 15d in ref 42), a merged human butyrylcholinesterase (hBChE) inhibitor/cannabinoid receptor 2 (hCB2R) ligand and a therapeutic target for preventing learning impairments in Alzheimer’s disease (Figure 5B).^42^ The *baseline* multistep model failed to identify a synthetic route, while the *mixed fine-tuned* model predicts retrosynthetic disconnections similar to the literature route (Figure 5). Reagents were omitted from the literature route to focus on the core synthons. While the *mixed fine-tuned* model deviated by not reproducing the carbamate disconnection of **ADD** to benzyl-protected phenol **D1**, instead using the presynthesized phenyl carbamate **E2**, it proposed subsequent disconnections featuring the same cyclization, reduction, and S_N_Ar as the literature route to mutually predicted reactants **E1**, **F1**, **G1**, and **G2**. This further reaffirms the improved ring-breaking performance in multistep retrosynthesis of the *mixed fine-tuned* model, where the *baseline* model failed for the benzoimidazole scaffold in **ADD**.

These results demonstrate the capability of the *mixed fine-tuned* multistep model in suggesting tractable synthetic routes for newly discovered, complex, drug-like targets containing heterocycles. This highlights its potential as a tool for synthetic chemists, aiding them in designing synthetic routes toward novel heterocycle-containing therapeutics.

### Recently Developed Heterocycle Formation Reactions

To evaluate whether the *mixed fine-tuned* model could extrapolate to unknown disconnections, we extracted 1.5k heterocycle ring-forming reactions from 47 papers published in 2022 detailing new heterocycle formation methodologies (here referred to as the *Recent* data set). While the model could not predict the exact reported reactions, it generated chemically valid ring-breaking predictions for 30.4% of the molecules. This indicates that while many of the heterocycles formed were already synthetically accessible, the reported routes were potentially more efficient or greener than those already reported (Figure 6A). Interestingly, the routes suggested by our model often resembled the ground truth (Figure 6A­(i–iii)). For example, both the *mixed fine-tuned* model and literature suggested the same Friedländer synthesis for quinoline (Figure 6A­(i)). In the literature synthesis, there is an additional oxime intermediate;^43^ however, the *mixed fine-tuned* model’s prediction follows the direct approach previously taken for trifluoromethane-substituted quinolines by Jiang et al.^44^

**Figure 6:**
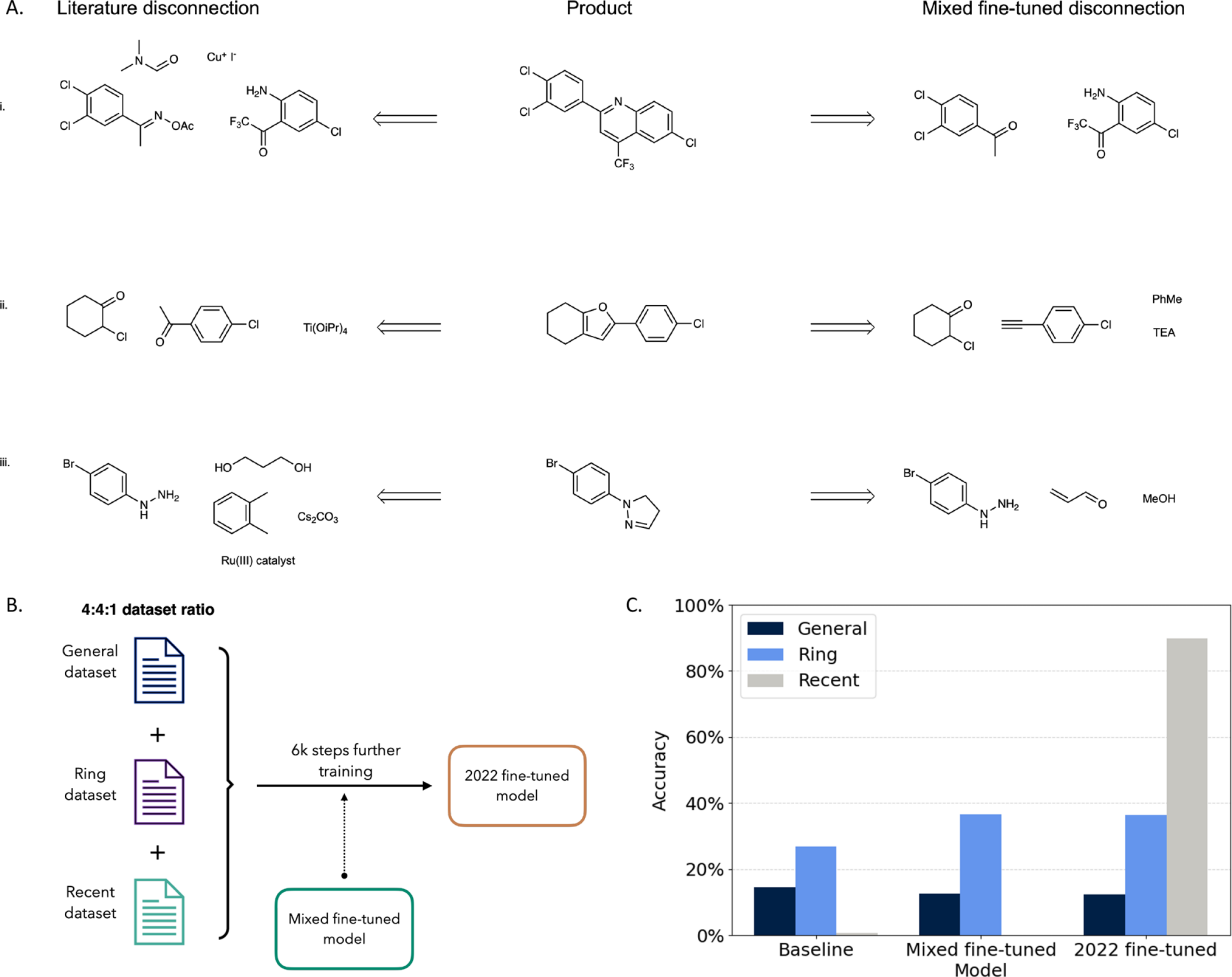
Recent reaction prediction. (A) Example valid predictions of the *mixed fine-tuned* model on the *Recent* test set. (B) The further fine-tuning approach: the *mixed fine-tuned* model is further trained on all three data sets. (C) Top-1 accuracy for the *baseline*, *mixed fine-tuned*, and *further fine-tuned* model on *General*, *Ring*, and *Recent* test sets. Reactant-only accuracy is reported for the *Ring* and *Recent* test sets.

Although the *mixed fine-tuned* model found valid ring-breaking disconnections for almost a third of the molecules in the *Recent* test set, when compared to the *Ring* test set, this proportion is lower by 30%. Therefore, this indicates that the *Recent* test set includes a higher number of heterocycles unknown to our model and is therefore considered synthetically inaccessible. If the model was trained on those new heterocycle formations, it could potentially explore a new region of the chemical space. To address this, we further trained the *mixed fine-tuned* model using the *Recent* data set. This updated *2022 fine-tuned* model was trained on the three data sets—*General*, *Ring*, and *Recent*—for another 6000 steps starting from the *mixed fine-tuned* model (Figure 6B). The top-1 accuracy of this *2022 fine-tuned* model is shown in Figure 6C. This updated *2022 fine-tuned* model exhibited only a slight decrease in accuracy on the *General* and *Ring* test sets while showing an increased top-1 reactant accuracy on the *Recent* test set (89.9%). This illustrates that the model can be fine-tuned to incorporate new reaction data without significantly compromising performance on previously learned tasks. While we used a small data set of heterocycle formations here, this approach could be applied to a larger data set or reaction data for different reaction classes of interest.

## Conclusion

In this work, we compared four different transfer learning approaches: fine-tuning, multitask learning, mixed fine-tuning, and ensemble decoding. Our aim was to improve the performance of seq2seq retrosynthesis prediction models for ring-breaking disconnections. We have found that mixed fine-tuning performs best overall, with a short training time and top-1 reactant accuracy for ring formations increased by 10% compared to the *baseline* model and a barely decreased accuracy on other reaction classes. The accuracy for ring formations is comparable to the template-based model we trained based on “Ring Breaker”; however, the *mixed fine-tuned* model vastly outperforms the template-based model in other reaction classes. While the *fine-tuned* model performs best for ring formations, with top-1 reactant accuracy of 40.5%, its performance significantly drops for other reaction classes due to catastrophic forgetting. This makes it unusable for multistep retrosynthesis, which requires disconnection of both rings and linear intermediates. We have also introduced a new metric, the “ring-breaking round-trip accuracy”, to assess the performance of the models for ring-breaking disconnections. By comparing the round-trip accuracy and ring-breaking round-trip accuracy of the *baseline* and *mixed fine-tuned* models, we have shown that both models suggested viable disconnections for a similar proportion of molecules. However, the key improvement in the *mixed fine-tuned* model was the type of disconnection that was suggested. While the *baseline* model suggests common reactions, such as protections/deprotections or functional group interconversions, which were either unnecessary or better suited earlier in the synthetic route, the *mixed fine-tuned* model favored ring formation reactions, with 62.1% of disconnections being ring-breaking round-trip accurate.

We then showcased the practical utility of the *mixed fine-tuned* model by using it for multistep retrosynthesis of two newly discovered, complex drug-like compounds containing heterocycles. This illustrates how the model can be used to assist synthetic and medicinal chemists, aiding them in designing synthetic routes toward novel heterocycle-containing therapeutics.

Finally, we have introduced a method for further fine-tuning the model on the basis of additional reaction data. By using this further mixed fine-tuning, we have substantially improved the model’s top-1 reactant accuracy on ring formation reactions published in 2022 from 0% to 89.9% without significantly compromising performance for older ring formation reactions or other reaction classes. While this approach has been applied to a small data set of less than 1.5k heterocycle formations, it has the potential to be scaled up for a larger data set or a different reaction class.

## Supplementary Material



## Data Availability

The General data set (based on USPTO), the ring formation reactions derived from CJHIF, and the Recent data set are available at: https://github.com/duartegroup/Het-retro. The source code for single-step model training and inference is available at: https://github.com/duartegroup/Het-retro.

## Notes

Details on the hyperparameters used in the transformer and template-based models; analysis of the chemical space covered by the data sets; overview of the different splitting strategies and their influence on performance; extended performance analysis across different models; and discussion on the metrics used to evaluate performance (PDF)

## ∥

Laboratory of Artificial Chemical Intelligence (LIAC), École Polytechnique Fédérale de Lausanne (EPFL), Lausanne, Switzerland

## ∇

SandboxAQ, Palo Alto, California 94301, United States
