# Supplementary material for: Transfer Learning for Heterocycle Retrosynthesis
Source: J Chem Inf Model. 2025 Jul 29;65(15):7851–61. doi: 10.1021/acs.jcim.4c02041 (PMC12344764; doi:10.1021/acs.jcim.4c02041)
Supplement: Supplementary file 1 [file ci4c02041_si_001.pdf]

Supporting Information

Transfer Learning for Heterocycle Retrosynthesis

Ewa Wieczorek, Joshua W Sin, Sara Tanovic, Matthew T. O. Holland, Liam Wilbraham, Victor Sebastián-Pérez, Anthony Bradley, Dominik Miketa, Paul E Brennan, Fernanda Duarte

Contents

S1 Transformer model hyperparameters **S2**

S2 Detailed dataset breakdown **S3**

S3 Ring dataset **S4**

S4 Chemical space of the data **S5**

S5 Metrics used to assess single-step model performance **S7**

S6 Mixed fine-tuning optimisation **S8**

S7 Effect of dataset splitting **S10**

S8 Evaluation of single-step models **S11**

S9 Forward reaction prediction model **S12**

S10 Recent reaction dataset **S13**

S1 Transformer model hyperparameters

The transformer models were trained using OpenNMT-py.[1]

The data was first preprocessed:

```
onmt_preprocess -train_src ${DATASET}/tgt-train.txt
${DATASET_TRANSFER}/product-train.txt -train_tgt ${DATASET}/src-train.txt
${DATASET_TRANSFER}/reactant-train.txt -train_ids general ring
-valid_src ${DATASET_TRANSFER}/product-valid.txt -valid_tgt
${DATASET_TRANSFER}/reactant-valid.txt -save_data ${DATADIR}/mft_retro
-src_seq_length 3000 -tgt_seq_length 3000 -src_vocab_size 3000
-tgt_vocab_size 3000 -share_vocab
```

The following command was used to train the *mixed fine-tuned* model:

```
onmt_train -data $DATADIR/mft_retro  -save_model
$MODELDIR/mft_model -save_checkpoint_steps 1000
-data_ids general ring --data_weights $WEIGHT1 $WEIGHT2
-seed $SEED -gpu_ranks 0   -train_steps 256000 -param_init 0
-param_init_glorot  -train_from $MODELDIR/retro_model_pretrained.pt
-max_generator_batches 32   -batch_size 6144 -batch_type tokens
-normalization tokens -max_grad_norm 0 -accum_count 4
-optim adam -adam_beta1 0.9 -adam_beta2 0.998 -decay_method noam
-warmup_steps 8000 -learning_rate 2 -label_smoothing 0.0
-layers 4 -rnn_size 384 -word_vec_size 384
-encoder_type transformer -decoder_type transformer
-dropout 0.1 -position_encoding -share_embeddings
-global_attention general -global_attention_function softmax
-self_attn_type scaled-dot -heads 8 -transformer_ff 2048
--tensorboard -tensorboard_log_dir $DATADIR/logs
```

The -train_from argument was used for *fine-tuned* and *mixed fine-tuned* models. The -data_weights argument was used for *multi-task* and *mixed fine-tuned* models.

The following command was used to make predictions:

```
onmt_translate -model $MODELDIR/retro_mft_model.pt
-src $DATADIR/ring_dataset/product-test.txt
-output $PREDDIR/retro_mft_ring_predictions_top5.txt
-n_best 5 -beam_size 5 -max_length 300 -batch_size 64 -gpu 0
```

S2 Detailed dataset breakdown

Table S1: Training, validation and test set sizes for each dataset used in this study.

Dataset	Train	Validation	Test
General	1,090,034	60,430	60,548
Ring	148,694	8,260	8,262
Random Ring	148,749	8,276	8,191
Recent	1,180	147	148

S3 Ring dataset

To form the *Ring* dataset, ring formation reactions were extracted from both CJHIF and Pistachio.[2, 3] Both datasets first underwent a series of standardisations and validity filters. The reactions were canonicalised and duplicate entries removed together with reactions already present in USPTO. Reactions with more than one product were filtered out. Reagents in CJHIF were converted into the SMILES format with Chemical Identifier Resolver (https://cactus.nci.nih.gov).

For Pistachio, the ring formation reactions were extracted based on the assigned reaction superclass "Heterocycle formations" (82,486 reactions). For CJHIF, the reactions were extracted based on the difference in the number of rings in the product and in the reactants as calculated by RDKit (83,623 reactions). Reactions from the two sources were then combined and duplicates dropped, resulting in the final *Ring* dataset containing 165,216 reactions.

S4 Chemical space of the data

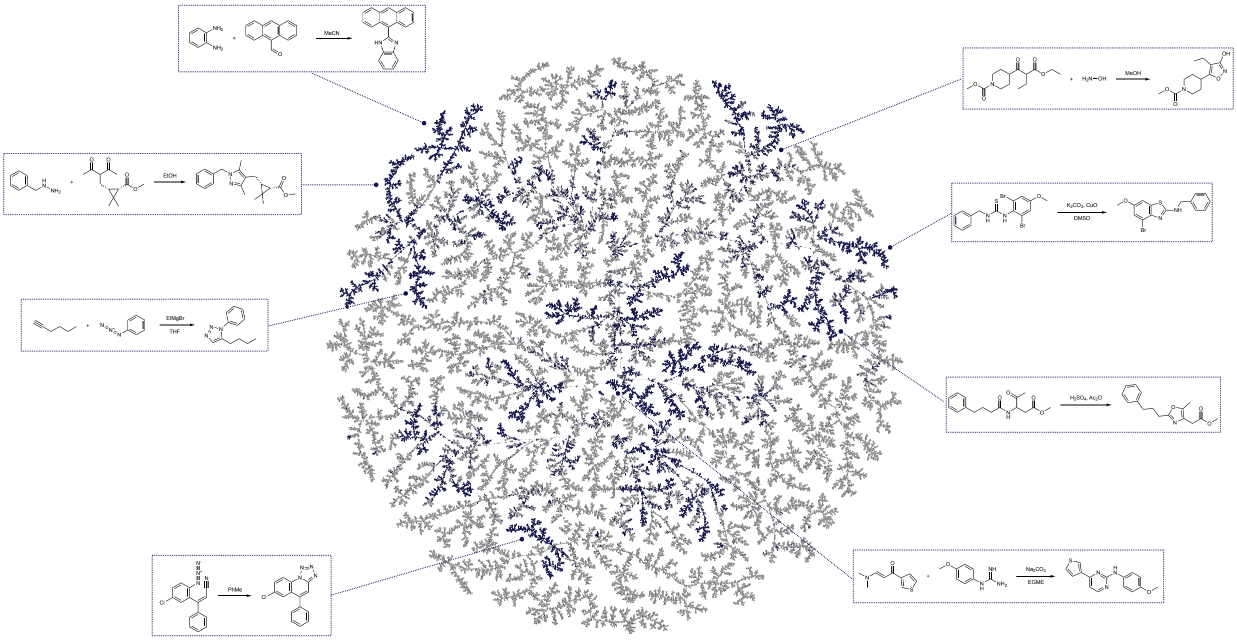

Figure S1: Tmap of the reaction data used for transfer learning: *General* dataset in grey and *Ring* dataset in navy. Representative reactions are shown for some of the heterocycle formation clusters. Rxnfps[4] of the reactions were used to create the tmap.

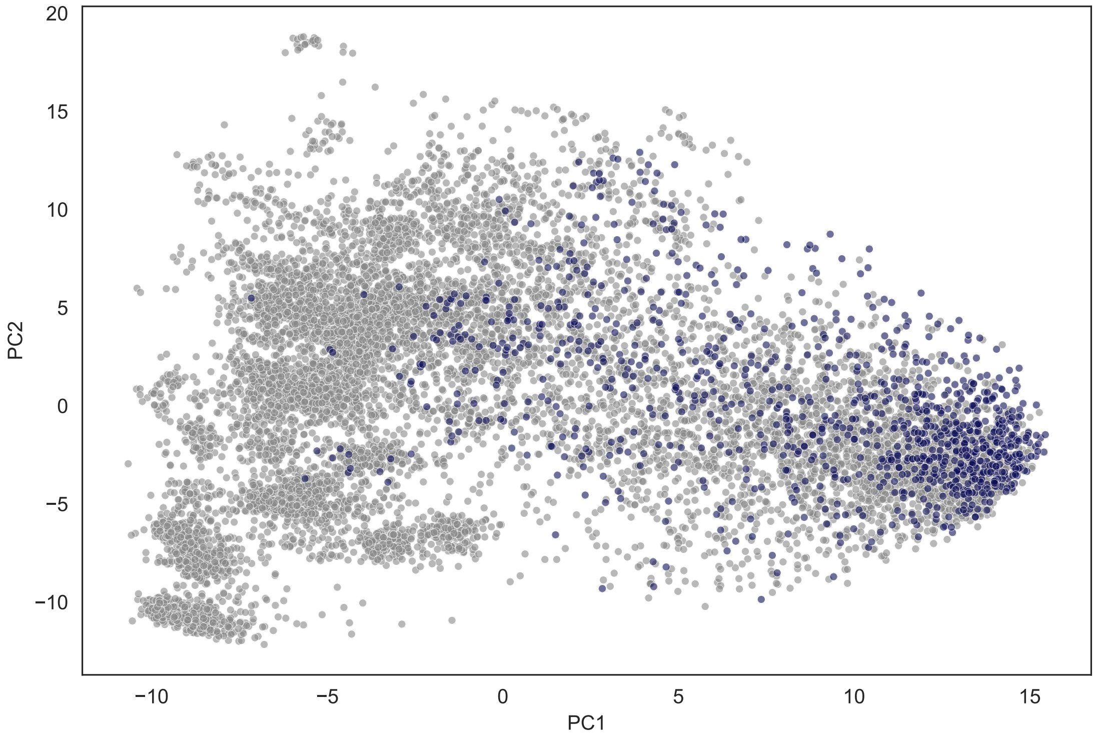

Figure S2: PCA of the reaction data used for transfer learning: a 10k random subset of the *General* dataset in grey and a 1k random subset of the *Ring* dataset in navy. Rxnfps[4] of the reactions were used to create the PCA.

S5 Metrics used to assess single-step model performance

Top-N accuracy:

Top-N accuracy quantifies the proportion of test set reactions for which the ground truth disconnection is present in the top-N predictions of the model, i.e. at least one of the N most likely predictions given by the model matches the literature disconnection in the test set.

Round-trip accuracy:

Round-trip accuracy quantifies the proportion of (top-N) predictions that are chemically valid, i.e. the predicted precursors are expected to react to form the original product (as judged by the top-1 prediction of the forward reaction prediction model).

Ring-breaking round-trip accuracy:

Ring-breaking round-trip accuracy quantifies the proportion of (top-N) predictions that are both round-trip accurate (see above) and ring-breaking. A disconnection is judged to be ring-breaking if the number of rings in the product is greater than the number of rings in the reactants (as given by RDKit). Only the predicted precursor molecules with at least one mapped atom (after atom-mapping with rxnmapper[5]) are treated as reactants.

Coverage:

Coverage quantifies the proportion of test set reaction products for which at least one chemically valid prediction is found among the top-N predictions.

S6 Mixed fine-tuning optimisation

We studied the effect of dataset weight ratio and number of fine-tuning steps on the performance of the *mixed fine-tuned* model.

The dataset weights control the proportion of reactions from each dataset (General and Ring) included in each training batch. We trained five different models with dataset ratios ranging from 9 General: 1 Ring to 1 General: 9 Ring (Figure S3). Increasing the ratio of heterocycle formations improves the model performance on ring-forming reactions but decreases it for other reaction classes. The optimal dataset ratio was found to be 1:1, demonstrating a significant improvement over other partitions while maintaining a similar accuracy for predicting general reactions (baseline model). Consequently, we utilized the 1:1 dataset ratio for mixed fine-tuning.

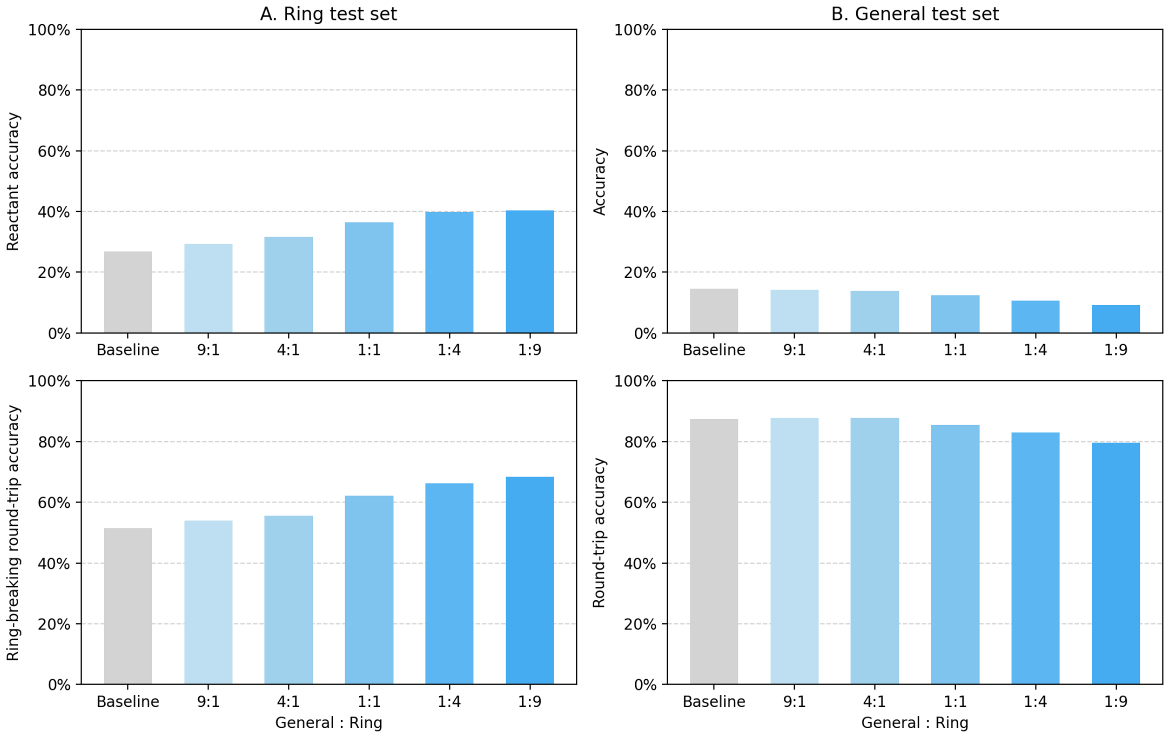

Figure S3: Effect of dataset weight ratio on model performance in mixed fine-tuning. Top-1 reactant accuracy and proportion of valid ring-breaking top-1 predictions are shown for the Ring test set. Top-1 accuracy and round-trip accuracy are shown for the General test set.

Next, we investigated the effect of the number of fine-tuning steps (Figure S4). Fine-tuning for even 2000 steps shows an improved performance compared to the baseline model. However, further improvements are marginal, with around 1% improvement for every additional 2,000 steps. Beyond 6,000 steps, the reactant accuracy fluctuates around 36.5%, indicating that further fine-tuning does not enhance the model performance. Thus, we conclude that fine-tuning for 6,000 steps with a 1:1 dataset weight ratio yields the most optimal results.

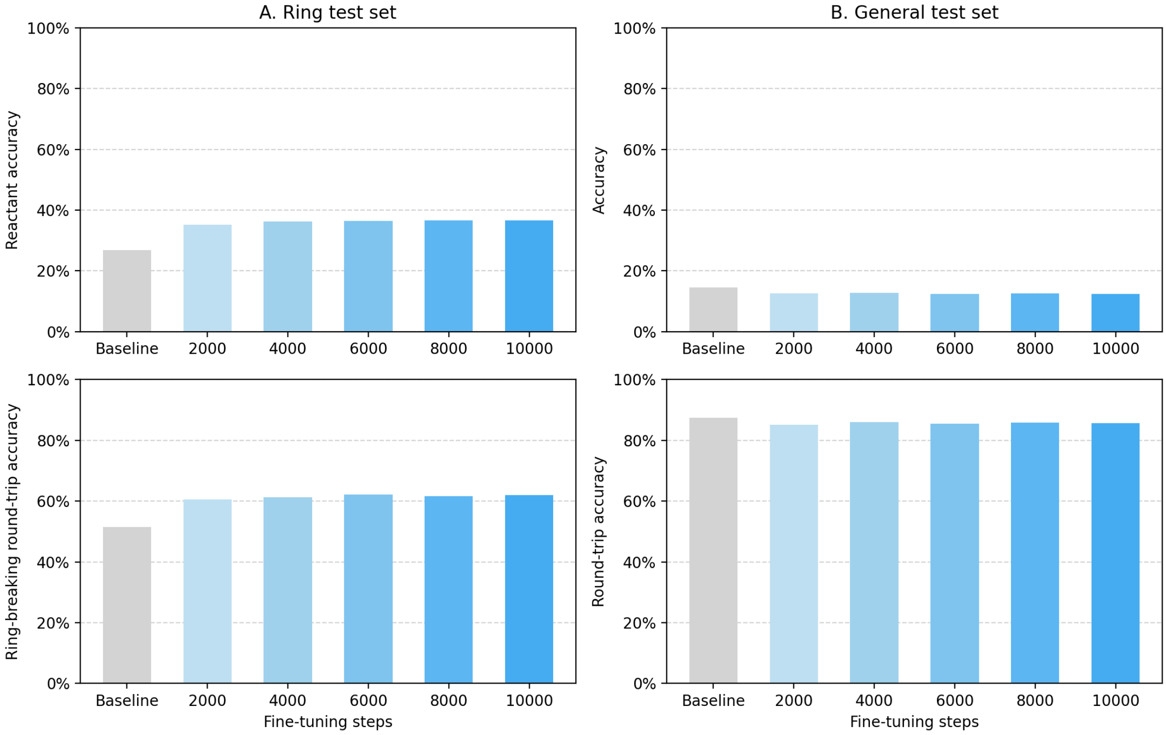

Figure S4: Effect of the number of fine-tuning steps on model performance in mixed fine-tuning. Top-1 reactant accuracy and proportion of valid ring-breaking top-1 predictions are shown for the Ring test set. Top-1 accuracy and round-trip accuracy are shown for the General test set.

S7 Effect of dataset splitting

While the importance of splitting based on Tanimoto similarity of the reaction product has been demonstrated by Lee *et al.*[6] for forward reaction prediction, most retrosynthesis prediction models still use a random train/test split. In this work, we have used Tanimoto similarity to split the Ring dataset into train, validation and test sets when comparing the different domain adaptation methods. However, here we examine the effect of dataset splitting by comparing the performance of the *mixed fine-tuned* model trained on a randomly split dataset as shown in Figure S5.

Our results show that the performance on the General test set is comparable to the model trained on Tanimoto split data, achieving an accuracy of 12.6% and round-trip accuracy of 85.7%. As the Ring test sets are different, a direct comparison between accuracies is not possible; however, we can assess the improvement between the *baseline* and *mixed fine-tuned* model. As expected, this improvement in reactant-accuracy is greater when the model is trained on randomly split data, increasing from 19.9% reactant-accuracy of the *baseline* model to 35.0% for the *mixed fine-tuned* model. In comparison, when the model is trained on Tanimoto split data, the accuracy increases from 26.9% to 36.5%. This supports Lee's conclusion that using a random split overstates the model's predictive ability.

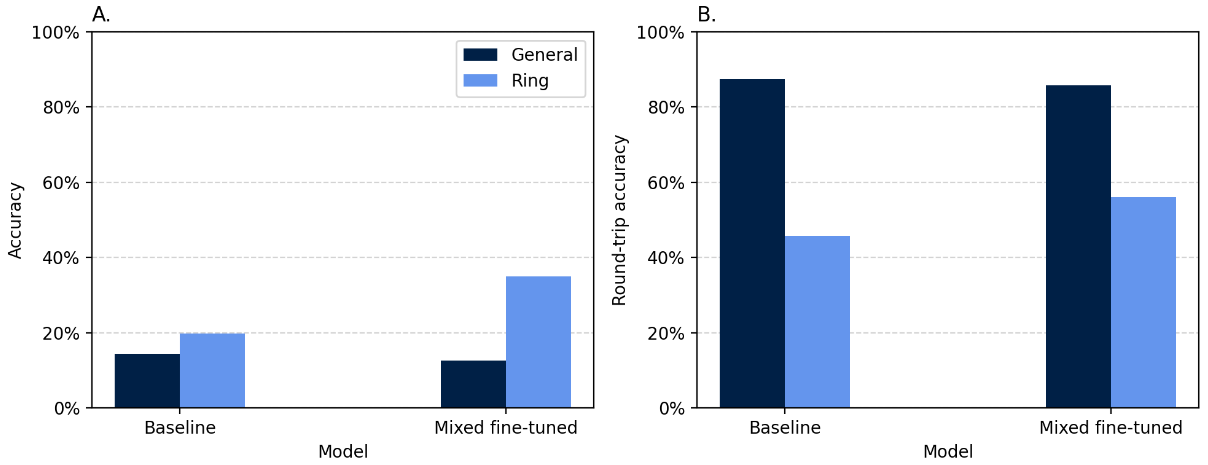

Figure S5: Performance of the *mixed fine-tuned* model trained on randomly split Ring dataset compared to the *baseline* model: (A) Top-1 accuracy on the General test set and top-1 reactant accuracy on the randomly split Ring test set and (B) top-1 round-trip accuracy on the General test set and proportion of valid ring breaking top-1 predictions for the Ring test set.

S8 Evaluation of single-step models

Table S2 includes top-1/3/5 reactant accuracy, round-trip accuracy, coverage and ring-breaking round-trip (r-t) accuracy for all single-step models when tested on the *Ring* test set.

Table S2: Metrics to assess performance on Ring test set

Model	Reactant accuracy			Round-trip accuracy			Coverage			Ring-breaking r-t accuracy		
	top-1	top-3	top-5	top-1	top-3	top-5	top-1	top-3	top-5	top-1	top-3	top-5
Baseline	26.9%	35.9%	38.9%	75.9%	73.7%	72.0%	75.9%	84.7%	86.9%	51.4%	49.7%	48.2%
Ring-only	37.2%	47.5%	50.9%	63.5%	59.6%	56.6%	63.5%	73.2%	76.4%	59.5%	56.6%	53.9%
Multi-task	35.0%	47.1%	50.4%	77.9%	76.5%	75.1%	77.9%	86.3%	88.2%	58.5%	57.6%	56.5%
Fine-tuned	40.5%	51.5%	54.5%	72.1%	70.4%	69.1%	72.1%	81.7%	84.4%	69.5%	68.1%	67.0%
Mixed fine-tuned	36.5%	48.6%	51.6%	74.6%	73.3%	71.8%	74.6%	84.4%	86.9%	62.1%	61.3%	59.9%
Ensemble decoding	36.3%	48.5%	52.7%	71.8%	69.7%	68.0%	71.8%	82.2%	84.7%	63.9%	62.1%	60.2%
Template-based	37.5%	53.8%	60.3%	64.8%	58.1%	52.4%	64.8%	76.7%	79.9%	64.8%	58.1%	52.4%

Table S3 includes top-1/3/5 accuracy (calculated for both reactants and reagents), reactant accuracy, round-trip accuracy and coverage for all single-step models when tested on the *General* test set.

Table S3: Metrics to assess performance on General test set

Model	Top-N accuracy			Reactant accuracy			Round-trip accuracy			Coverage		
	top-1	top-3	top-5	top-1	top-3	top-5	top-1	top-3	top-5	top-1	top-3	top-5
Baseline	14.5%	21.5%	23.7%	26.4%	34.3%	36.8%	87.4%	85.4%	83.7%	87.4%	93.4%	94.7%
Ring-only	0.4%	0.7%	0.8%	2.0%	2.7%	3.0%	21.8%	20.0%	18.9%	21.8%	29.7%	33.5%
Multi-task	13.6%	20.6%	22.7%	26.1%	33.9%	36.5%	87.5%	86.0%	84.5%	87.5%	93.4%	94.7%
Fine-tuned	2.7%	4.6%	5.3%	11.4%	16.1%	17.8%	52.6%	49.7%	48.4%	52.6%	67.1%	71.7%
Mixed fine-tuned	12.5%	19.1%	21.0%	24.8%	32.7%	35.2%	85.4%	83.2%	81.3%	85.4%	92.2%	93.7%
Ensemble decoding	9.3%	14.2%	15.6%	22.7%	30.1%	32.3%	77.9%	75.7%	74.1%	77.9%	87.5%	89.9%
Template-based	0.6%	0.6%	0.6%	3.2%	3.7%	3.8%	28.2%	22.1%	17.7%	28.2%	34.1%	35.5%

S9 Forward reaction prediction model

Round-trip accuracy and coverage were calculated based on the predictions of a forward reaction prediction model. This model was trained using multi-task learning following the approach of Pesciullesi *et al.*[7] Table S4 contains the top-1/3/5 accuracies of this multi-task model together with the same metrics for a baseline forward reaction prediction model.

Table S4: Accuracy of the forward reaction prediction models on the General and Ring test sets.

	Test set					
Model	General			Ring		
	top-1	top-3	top-5	top-1	top-3	top-5
Baseline	78.0%	84.5%	86.7%	61.3%	76.9%	79.4%
Multi-task	78.4%	86.1%	87.3%	74.3%	88.3%	89.8%

S10 Recent reaction dataset

The reactions forming the *Recent* dataset were extracted from the reactant scope sections of the following articles:

Z.-H. Wang, L.-W. Shen, P. Yang, Y. You, J.-Q. Zhao, W.-C. Yuan, J. Org. Chem., 2022, 87, 5804-5816.

Z. Liu, S. Zhong, X. Ji, G.-J. Deng, H. Huang, Org. Lett., 2022, 24, 349-353.

Z. Li, K. Qiu, X. Yang, W. Zhou, Q. Cai, Org. Lett., 2022, 24, 2989-2992.

Z. Alikhani, A. G. Albertson, C. A. Walter, P. J. Masih, T. Kesharwani, J. Org. Chem., 2022, 87, 6312-6320.

Y.-H. Ma, F.-X. Meng, R.-N. Wang, Y.-X. Fan, Q.-Q. Su, J.-Y. Du, Synthesis, 2022, 54, 499-505.

Y.-C. Liu, P. Chen, X.-J. Li, B.-Q. Xiong, K.-W. Tang, P.-F. Huang, J. Org. Chem., 2022, 87, 4263-4272.

Y. Zheng, Y. Long, H. Gong, J. Xu, C. Zhang, H. Fu, X. Zheng, H. Chen, R. Li, Org. Lett., 2022, 24, 3878-3883.

Y. Yamaguchi, Y. Seino, A. Suzuki, Y. Kamei, T. Yoshino, M. Kojima, S. Matunaga, Org. Lett., 2022, 24, 2441-2445.

Y. V. Ostapiuk, M. Shehedyn, O. V. Barabash, B. Demydchuk, S. Batsyts, C. Herzberger, A. Schmidt, Synthesis, 2022, 54, 732-740.

X. Jin, L. Xing, D. D. Deng, Y. Yan, Y. Fu, W. Dong, J. Org. Chem., 2022, 87, 1541-1544.

W. Li, R. Shi, S. Chen, X. Zhang, W. Peng, S. Chen, J. Li, X.-M. Xu, Y.-P. Zhu, X. Wang, J. Org. Chem., 2022, 87, 3014-3024.

T. Yang, H. Li, Z. Nie, M.-d. Su, W.-p. Luo, Q. Liu, C.-C. Guo, J. Org. Chem., 2022, 87, 2797-2808.

S. Zhang, Q. Zhang, M. Tang, J. Org. Chem., 2022, 87, 3845-3850. R. Zhang, M. Sun, Q. Yan, X. Lin, X. Li, X. Fang, H. H. Y.

Sung, J. D. Williams, J. Sun, Org. Lett., 2022, 24, 2359-2364.

Q. Guo, J. Chen, G. Shen, G. Lu, X. Yang, Y. Tang, Y. Zhu, S. Wu, B. Fan, J. Org. Chem., 2022, 87, 540-546.

M. Vadivelu, A. A. Raheem, J. P. Raj, J. Elangovan, K. Karthikeyan, C. Praveen, J. Sun, Org. Lett., 2022, 24, 2798-2803.

M. V. Il'in, A. A. Sysoeva, A. S. Novikov, D. S. Bolotin, J. Org. Chem., 2022, 87, 4569-4579.

M. Maji, I. Borthakur, S. Srivastava, S. Kundu, J. Org. Chem., 2022, 87, 5603-5616.

M. Baidya, S. Mallick, S. D. Sarkar, Org. Lett., 2022, 24, 1274-1279.

M. B. Reddy, K. Prasanth, R. Anandhan, Org. Lett., 2022, 24, 3674-3679.

M. A. Ansari, G. Kumar, M. S. Singh, Org. Lett., 2022, 24, 2815-2820.

L. Ren, J. Luo, L. Tan, Q. Tang, J. Org. Chem., 2022, 87, 3167-3176.

L. Liu, J. Lin, M. Pang, H. Jin, X. Yu, S. Wang, Org. Lett., 2022, 24, 1146-1151.

K. Sakai, K. Oisaki, M. Kanai, Org. Lett., 2022, 24, 3325-3330.

K. Ishihara, T. Shioiri, M. Matsugi, Synlett, 2022, 33, 781-784.

K. H. Min, N. Iqbal, E. J. Cho, Org. Lett., 2022, 24, 989-994.

K. Gnyawali, P. T. K. Arachchige, C. S. Yi, Org. Lett., 2022, 24, 218-222.

J.-L. Liu, W. Wang, X. Qi, X.-F. Wu, Org. Lett., 2022, 24, 2248-2252.

J. Ying, T. Liu, Y. Liu, J.-P. Wan, Org. Lett., 2022, 24, 2393-2398.

J. Talvitie, I. Alanko, E. Bulatov, J. Koivula, T. Pöllänen, J. Helaja, Org. Lett., 2022, 24, 274-278.

J. Li, Y. Liu, Z. Chen, J. Li, j. Li, X. Ji, L. Chen, Y. Huang, Q. Liu, Y. Li, J. Org. Chem., 2022, 87, 3555-3566.

J. K. Laha, M. K. Hunjan, J. Org. Chem., 2022, 87, 2315-2323.

J. Jiao, P. Wang, F. Xiao, Z. Zhang, Synlett, 2022, 33, 569-574.

J. Hou, G. Yang, Z. Chai, J. Org. Chem., 2022, 87, 453-463.

J. Dong, J. Hu, X. Liu, S. Sun, L. Bao, M. Jia, X. Xu, J. Org. Chem., 2022, 87, 2845-2852.

H. Shui, Y. Zhong, L. Ouyang, N. Luo, R. Luo, Synthesis, 2022, 54, 2876-2884.

H. Guo, L. Tian, Y. Liu, J.-P. Wan, Org. Lett., 2022, 24, 228-233.

G. E. Bell, J. W. B. Fyfe, E. M. Israel, A. M. Z. Slawin, M. Campbell, A. J. B. Watson, Org. Lett., 2022, 24, 3024-3027.

F. S. Movahed, S. W. Foo, S. Mori, S. Ogawa, S. Saito, J. Org. Chem., 2022, 87, 243-257.

F. Lu, Y. Chen, X. Song, C. Yu, T. Li, K. Zhang, C. Yao, J. Org. Chem., 2022, 87, 6902-6909.

D. Zhuang, T. Gatera, Z. An, R. Yan, Org. Lett., 2022, 24, 771-775.

D. Jankovič, M. Virant, M. Gazvoda, J. Org. Chem., 2022, 87, 4018-4028.

C. Shan, J. Xu, L. Cao, C. Liang, R. Cheng, X. Yao, M. Sun, J. Ye, Org. Lett., 2022, 24, 3205-3240.

B. Ramesh, M. Jeganmohan, J. Org. Chem., 2022, 87, 6902-6909.

B. Lin, Y. Yao, Y. Huang, Z. Weng, Org. Lett., 2022, 24, 2055-2058.

A. K. Guin, R. Mondal, G. Chakraborty, S. Pal, N. D. Paul, J. Org. Chem., 2022, 87, 7106-7123.

J. Zhang, Y. Zhang, J. Zhang, Q. Wu, H. Yang, Synlett, 2022, 33, 264-268.

References

(1) OpenNMT-py.

(2) Jiang, S.; Zhang, Z.; Zhao, H.; Li, J.; Yang, Y.; Lu, B.-L.; Xia, N. *IEEE Access* **2021**, *9*, 85071–85083.

(3) NextMove Software, Pistachio, 2022.

(4) Schwaller, P.; Probst, D.; Vaucher, A. C.; Nair, V. H.; Kreutter, D.; Laino, T.; Reymond, J.-L. *Nature Machine Intelligence* **2021**, *3*, 144–152.

(5) Schwaller, P.; Hoover, B.; Reymond, J.-L.; Strobelt, H.; Laino, T. *Science Advances* **2021**, *7*, eabe4166.

(6) Kovács, D. P.; McCorkindale, W.; Lee, A. A. *Nat Commun* **2021**, *12*, 1695.

(7) Pesciullesi, G.; Schwaller, P.; Laino, T.; Reymond, J.-L. *Nat Commun* **2020**, *11*, 4874.
